# Quality of life among women of reproductive age during the accumulation of multiple chronic conditions

**DOI:** 10.1007/s11136-026-04262-4

**Published:** 2026-05-03

**Authors:** Mohammad R. Baneshi, Annette Dobson, Gita D. Mishra

**Affiliations:** Australian Women and Girls’ Health Research Centre, School of Public Health, Faculty of Health, Medicine, and Behavioural Sciences, The University of Queensland, Level 2 Edith Cavell Building, Herston Square, Herston, QLD 4006 Australia

**Keywords:** HRQOL, Longitudinal, Reproductive age, Temporal association

## Abstract

**Purpose:**

We examined the temporal association between the number of chronic conditions and health-related quality of life (HRQOL) among women of reproductive age and assessed how individual conditions affected specific HRQOL dimensions.

**Methods:**

Data from 9323 women born between 1973 and 1978 who participated in the Australian Longitudinal Study on Women’s Health and had no chronic health condition at the baseline survey were analysed. Linear mixed-effects models examined the temporal association between the number of conditions and SF-36 HRQOL and also compared HRQOL between women with and without six conditions: musculoskeletal disorders, diabetes, asthma, endometriosis, uterine fibroids, and cancer.

**Results:**

Women who remained free of conditions during ages 18 to 49 maintained stable physical component scores (PCS) and improved mental component scores (MCS). Accumulating conditions was associated with declining scores, more in PCS than MCS. The largest and smallest decline per an additional condition was observed in role physical (6.5 points; 95% CI 6.0, 7.0) and mental health (1.2 points; 0.9, 1.5). Within-group analysis found that all conditions were associated with PCS decline. Effects on MCS varied by condition and outcome. Between-group comparisons revealed that women with musculoskeletal disorders, diabetes, or asthma had lower PCS and MCS scores than women without those conditions. Endometriosis, uterine fibroids, and cancer showed smaller and more dimension-specific reductions.

**Conclusion:**

Women who did not develop chronic conditions maintained their PCS scores and had improvements in MCS scores. This underscores the value of preventing chronic conditions in early and mid-adulthood for maintaining physical and mental well-being.

**Supplementary Information:**

The online version contains supplementary material available at 10.1007/s11136-026-04262-4.

## Introduction

Multimorbidity, defined as the coexistence of two or more chronic conditions in the same individual, is a growing global public health concern [1], driven by population ageing and improved survival from chronic diseases [2]. Therefore, understanding the broad impact of multimorbidity has become important.

Multimorbidity is associated with poorer health outcomes, higher health service use and increased hospitalisation [3]. Cassell et al. found that in England, patients with multimorbidity accounted for 52.9% of GP consultations, 78.7% of prescriptions, and 56.1% of hospital admissions [3].

Another major consequence of multimorbidity is poorer health-related quality of life (HRQOL) [4]. HRQOL is a key indicator for evaluating healthcare services [5], understanding patient satisfaction with care [6, 7], and predicting morbidity and mortality [8, 9].

Systematic reviews found that multimorbidity is associated with poorer HRQOL both in the midlife period (ages 40 to 65) [4] and in older populations [10]. A meta-analysis further confirmed this relationship, demonstrating a negative association between the number of chronic conditions and both physical health and mental health component scores [11].

Despite extensive research, key gaps remain. Most studies are cross-sectional [4, 10, 11], limiting insight into temporal associations that require longitudinal data [12]. Additionally, although women have higher multimorbidity [13] and poorer HRQOL [14] than men, most studies included both men and women or focused on older women [15, 16]. No longitudinal study has examined these associations in women of reproductive age, an earlier life stage that has been understudied in this context. It also remains unclear which chronic conditions most affect HRQOL. While counting the number of chronic conditions illustrates the overall burden, it may overlook how individual conditions influence specific dimensions of HRQOL [17].

The Australian Longitudinal Study on Women’s Health (ALSWH) provided a unique opportunity to examine the temporal effect of the number of chronic conditions and the individual contribution of specific conditions on HRQOL.

## Methods

### Study population

ALSWH is a prospective population-based study of four cohorts born in 1989-95, 1973-78, 1946-51 and 1921-26 [18]. The three oldest cohorts were randomly selected from all women in the Medicare database, which is the database of the national universal health insurance scheme that covers all citizens and permanent residents in Australia.

The current analyses are related to women born in 1973-78, capturing ages 18 to 49 years across nine surveys, which corresponded to the reproductive age period. The first survey was conducted in 1996, when the women were aged 18 to 23 years and the second in 2000. After that, the surveys were conducted in a three-year cycle, with the latest survey (Survey 9) completed in 2021 when the women were aged 43 to 49 years.

### List of chronic conditions

In ALSWH surveys, women were asked if they had been told by a doctor that they had specific conditions. For this paper, we focused on conditions with the highest burden of disease among Australian women of reproductive age [19]. Our list of conditions included two reproductive and four other chronic conditions: endometriosis; uterine fibroids; musculoskeletal disorders (included back pain, rheumatoid arthritis, osteoarthritis, and scoliosis); asthma; cancer (included malignant cancers but not skin cancers except for melanoma); and diabetes (included type 1 and 2 diabetes mellitus but not gestational diabetes).

Within this age group, several other conditions (e.g. dementia, eating disorders, stroke, and ischaemic heart disease) have very low prevalence, limiting their contribution to multimorbidity patterns. Depression or anxiety, although prevalent, were not included because of substantial conceptual overlap with the study outcome (i.e., HRQOL). Primary hypertension and hypercholesterolaemia were also not included, as these conditions are frequently treated as cardiovascular risk factors rather than chronic conditions in multimorbidity research. We therefore selected conditions that are both clinically relevant and sufficiently prevalent during the reproductive years.

In Surveys 1 and 2, women reported whether a doctor had ever diagnosed them with listed conditions. From Survey 3 onwards, the question referred to diagnoses or treatment in the past three years. Conditions were identified using ALSWH survey responses and linked administrative records. Additional data sources included hospital admissions; investigations, procedures, and medication prescriptions subsidised by the national health insurance scheme; assessments for aged care support; and medically certified causes of death. Details and the coverage periods are summarised in Supplementary Table S1.

### Study sample

Of the 14,247 women who participated in Survey 1, 13,501 consented to data linkage [20]. Among these, 11,114 were free of all six chronic conditions at the baseline survey. Of these, 9323 had at least two HRQOL measurements and were included in the study sample.

### Exposure variable

The exposure variable was the number of chronic conditions developed after the baseline survey and by Survey 9. Multimorbidity was defined as having a record of two or more of the six chronic conditions during this period.

### Outcome variables

The outcome was HRQOL, assessed across nine surveys using SF-36 questionnaire [21]. The questionnaire reflects individuals’ self-reported physical and mental functioning and perceived health status, rather than evaluations of healthcare quality or service delivery. It included eight dimensions scored from 0 to 100, with higher scores indicating better HRQOL: bodily pain (2 items), role limitations due to physical problems (4 items), general health (5 items, including the self-rated health item), physical functioning (10 items), role limitations due to emotional problems (3 items), mental health (5 items), social functioning (2 items), and vitality (4 items).

Two summary scores were also computed: the physical component score (PCS) and the mental component score (MCS). Both were derived from all eight dimensions, but with different weighting schemes. PCS placed greater weight on physical health dimensions, while MCS emphasised mental health dimensions. The mean (SD) for both scores was 50 (10).

### Ethical approval

The ALSWH has been granted ethics clearance by the Human Research Ethics Committees at the University of Newcastle (ref no. H-0760-0795) and the University of Queensland (ref no. 2004000224). The ALSWH also maintains institutional HREC approvals for external record linkage (approval numbers H-2011-0371 and 2012000132, respectively). In addition, access to national data collections is approved by the AIHW. Access to state and territory data collections is approved by an appropriate HREC for each jurisdiction. Details of ALSWH ethical approvals for linked administrative datasets are provided here: https://alswh.org.au/alswh-hrec-approvals/.

### Consent to participate

Informed consent was obtained from participants for each survey. ALSWH participants who decline health record linkage are excluded from linked data requests. Over 80% of all ALSWH participants have explicitly consented to record linkage. Since 2005, the responsible HRECs have approved opt-out consent; in addition, a waiver applies to unconsented participants who were deceased or lost to follow up before 2005. All methods were carried out in accordance with relevant guidelines and regulations. The authors declare that this work was in compliance with ethical standards.

### Statistical analysis

#### Descriptive statistics

For 13,501 women who consented to data linkage, the prevalence of each condition at Surveys 1 and 9 was reported. Among the 9323 women who formed the study sample, the incidence of conditions by Survey 9 was described.

#### Association between the number of chronic conditions and HRQOL

To illustrate the association between the number of chronic conditions and the trajectory of outcomes, first, average scores were plotted from ages 18 to 49, stratified by the number of conditions (i.e., after baseline survey and by Survey 9). In this analysis, the number of conditions was categorised as 0, 1, and 2 or more conditions (i.e., multimorbidity).

Secondly, separate linear regressions were used to estimate the mean differences in outcomes from Survey 1 to Survey 9 by the number of conditions (categorised as 0, 1, or 2 or more conditions). Thirdly, to examine the temporal associations between the number of chronic conditions and outcomes, separate linear mixed-effects models with an autoregressive correlation structure were fitted. The outcome was HRQOL at Survey ‘t’. The exposure variable was the time-dependent cumulative number of conditions after the baseline survey but before Survey ‘t’. All regression models were adjusted for place of residence, marital status, education, income, smoking, alcohol consumption, physical activity, body mass index, and menopausal status, and regression coefficients were estimated for both the main exposure and all covariates. The coefficient for the number of chronic conditions represented the average change in the outcome per additional condition.

#### Association between individual conditions and HRQOL

To illustrate the association between each condition and the trajectory of outcomes, separate plots depicting outcomes from the age of 18 to 49 were generated for women with and without each condition. The area between trajectory curves was quantified using the trapezoidal rule, where a larger area indicates a greater divergence in HRQOL between groups over time.

Separate linear regressions were fitted to estimate the mean differences in outcomes from Survey 1 to Survey 9 within each group of women with a given condition, as well as for women with no condition (i.e., within-group comparisons).

To further assess the association between individual conditions and HRQOL, between-group comparisons were conducted using linear mixed-effects models, with HRQOL at Survey ‘t’ as the outcome and the time-dependent status of conditions as the exposure variable. Linear mixed-effects models were used to estimate the marginal mean of outcomes for women with and without each condition, as well as their differences and 95% CI.

All linear and linear-mixed effects regression models described above were adjusted for the effects of place of residence, marital status, education, income, smoking, alcohol consumption, physical activity, Body Mass Index (BMI), and menopause status. Moreover, in all models, missing values were handled using a missing category.

To check the robustness of results, missing data were imputed 10 times using multiple imputation via chained equations, and the regression models described above were refitted. Using each imputed data set, the regression model was fitted, and coefficients and standard errors were estimated. Estimates across 10 imputed data sets were combined by applying Rubin’s rules.

#### Software

Analyses were performed using R, with the following packages: ‘tidyverse’ for data management, ‘ggplot2’ for visualisations, ‘mice’ for multiple imputation of missing data, and ‘nlme’ for mixed-effects models.

## Results

### Descriptive statistics

In the sample of 13,501 women who consented to data linkage, musculoskeletal disorders (10.1%) and asthma (7.9%) were most prevalent at Survey 1 (when women were aged 18 to 23 years), while four conditions had a prevalence below 1.0% (Table 1). By Survey 9 (when aged 43 to 49 years), musculoskeletal disorders (42.7%), endometriosis (13.9%), and asthma (11.5%) were most common. Multimorbidity increased from 1.5% at Survey 1 to 20.7% at Survey 9.

**Table 1:** Progression of chronic conditions over time in a cohort of Australian women born between 1973 and 1978

Condition	Women who consented to data linkage (*N* = 13501)		Study sample: women with no condition at baseline survey with at least two measurements of outcome (*N* = 9323)
	By survey 1 (aged 18 to 23 years)	By survey 9 (aged 43 to 49 years)	By survey 9 (aged 43 to 49 years)
Musculoskeletal disorders	1370 (10.1%)	5769 (42.7%)	3483 (37.4%)
Endometriosis	36 (0.3%)	1870 (13.9%)	1277 (13.7%)
Uterine fibroids	< 10 (0.0%)	756 (5.6%)	535 (5.7%)
Cancer	34 (0.3%)	660 (4.9%)	442 (4.7%)
Diabetes	71 (0.5%)	724 (5.4%)	399 (4.3%)
Asthma	1071 (7.9%)	1553 (11.5%)	339 (3.6%)
Distribution of the number of conditions			
0	11,114 (82.3%)	5779 (42.8%)	4608 (49.4%)
1	2193 (16.2%)	4931 (36.5%)	3315 (35.6%)
≥ 2 (i.e., multimorbidity)	194 (1.5%)	2791 (20.7%)	1400 (15.0%)
Distribution of other conditions that were not included in the analysis (because of overlap with outcome, or low prevalence)			
Depression or anxiety	2,934 (21.7%)	6,924 (51.2%)	4,835 (51.9%)
Ischemic heart disease	<10 (0.0%)	224 (1.6%)	129 (1.4%)
chronic obstructive pulmonary disease	< 10 (0.0%)	69 (0.5%)	22 (0.2%)
Stroke	< 10 (0.0%)	116 (0.8%)	65 (0.7%)
Eating disorders	52 (0.3%)	108 (0.7%)	77 (0.8%)
Dementia	< 10 (0.0%)	< 10 (0.0%)	< 10 (0.0%)

Among the 9323 women included in the study sample (i.e., those with no condition at baseline and at least two HRQOL reports), 37.4% developed musculoskeletal disorders and 13.7% developed endometriosis by Survey 9. The proportion of women who developed incident multimorbidity was 15.0% (Table 1).

Distribution of other chronic conditions, which are not considered in this study (because of overlap with outcome, or low prevalence), is also reported in Table 1.

At baseline survey, women who later developed multimorbidity (i.e., had two or more chronic conditions) had a less advantaged sociodemographic and health profile than those who remained free of chronic conditions (Table 2): fewer held a university degree (11.7% vs. 13.1%) obesity (8.3% vs. 4.0%) and current smoking (30.1% vs. 27.6%) were more common; high physical activity was less common (26.3% vs. 28.0%); and risky/very risky alcohol use was slightly higher (5.4% vs. 4.8%).

**Table 2:** Distribution of sociodemographic and health-related risk factors at baseline survey stratified by multimorbidity status at the end of survey (*N* = 9323)

Variable	Category	Number of conditions after baseline survey and by the end of the study		
		0 (*N* = 4608)	1 (*N* = 3315)	≥ 2 (*N* = 1400)
Education level	Below diploma	3306 (71.7%)	2427 (73.2%)	1000 (71.4%)
	Diploma	682 (14.8%)	468 (14.1%)	229 (16.4%)
	University degree	604 (13.1%)	398 (12.0%)	164 (11.7%)
	Missing	16 (0.3%)	22 (0.7%)	7 (0.5%)
Ability to manage on income	Impossible/ always difficult	698 (15.1%)	564 (17.0%)	249 (17.8%)
	Sometimes difficult	1448 (31.4%)	1075 (32.4%)	464 (33.1%)
	Not bad	1760 (38.2%)	1251 (37.7%)	477 (34.1%)
	Easy	691 (15.0%)	416 (12.5%)	204 (14.6%)
	Missing	11 (0.2%)	9 (0.3%)	6 (0.4%)
Area of residence	Major cities	2441 (53.0%)	1683 (50.8%)	699 (49.9%)
	Inner regional	1354 (29.4%)	1013 (30.6%)	457 (32.6%)
	Outer regional	669 (14.5%)	515 (15.5%)	199 (14.2%)
	Remote/ very remote	141 (3.1%)	101 (3.0%)	44 (3.0%)
	Missing	3 (0.1%)	3 (0.1%)	1 (0.1%)
Body mass index	Normal	3408 (73.9%)	2220 (67.0%)	913 (65.2%)
	Overweight	549 (11.9%)	496 (15.0%)	202 (14.4%)
	Obesity	183 (4.0%)	204 (6.2%)	116 (8.3%)
	Missing	99 (10.2%)	395 (11.9%)	169 (12.1%)
Smoking status	Never	2522 (54.7%)	1686 (50.9%)	706 (50.4%)
	Ex	623 (13.5%)	477 (14.4%)	221 (15.8%)
	Current	1274 (27.6%)	1010 (30.5%)	421 (30.1%)
	Missing	189 (4.1%)	142 (4.3%)	52 (3.7%)
Alcohol consumption	Low	2496 (54.2%)	1760 (53.1%)	681 (48.6%)
	None	386 (8.4%)	267 (8.1%)	115 (8.2%)
	Rare	1461 (31.7%)	1074 (32.4%)	517 (36.9%)
	Risky/ very risky	220 (4.8%)	179 (5.4%)	76 (5.4%)
	Missing	45 (1.0%)	35 (1.1%)	11 (0.8%)
Physical activity (at Survey 2)	Nil/ sedentary	310 (6.7%)	253 (7.6%)	119 (8.5%)
	Low	1117 (24.2%)	867 (26.2%)	380 (27.1%)
	Moderate	860 (18.7%)	610 (18.4%)	252 (18.0%)
	High	1290 (28.0%)	916 (27.6%)	368 (26.3%)
	Missing	1031 (22.4%)	669 (20.2%)	281 (20.1%)

For most covariates, the missingness rate at baseline was low (generally < 1%), with a higher rate for smoking (4.3%) and BMI (12.9%%). Among women who participated in Survey 9, missingness was around 5% for most covariates, except physical activity (12.0%) and alcohol use (12.7%).

### Association between the number of conditions and HRQOL

**Fig. 1:**
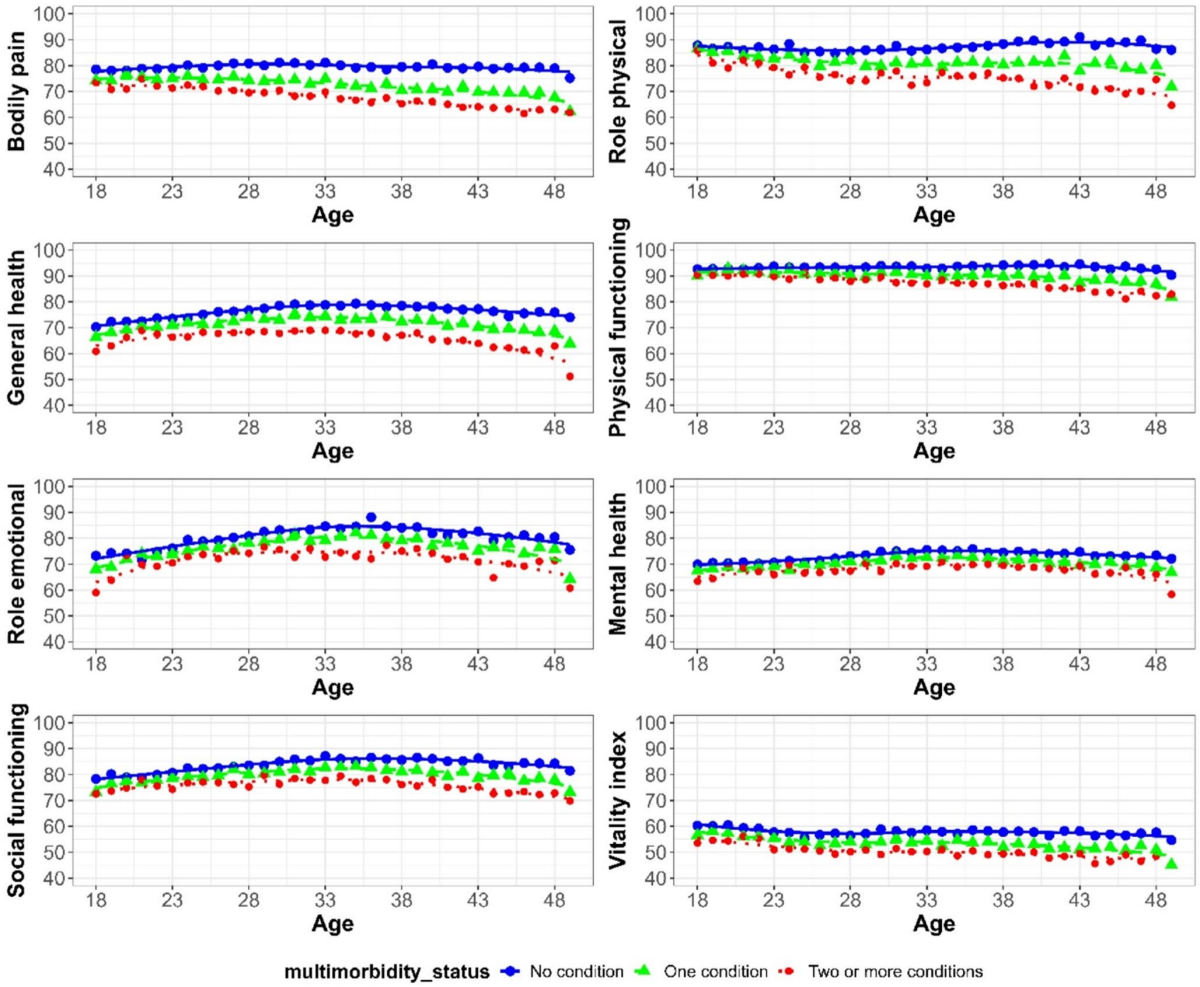
Average score of outcomes for women with no chronic condition at baseline survey, stratified by number of conditions by Survey 9 (*N* = 9323). Higher scores indicate better HRQOL

At age 18, outcome scores were highest in women without chronic conditions, lower among those with one condition, and lowest among those with multimorbidity (Fig. 1). From the age of 18 to 49, outcome scores remained largely stable among women without conditions, whereas most outcomes declined in those with multimorbidity. Across all groups, role emotional initially increased before showing a slight decline, and vitality declined gradually over time.

**Fig. 2:**
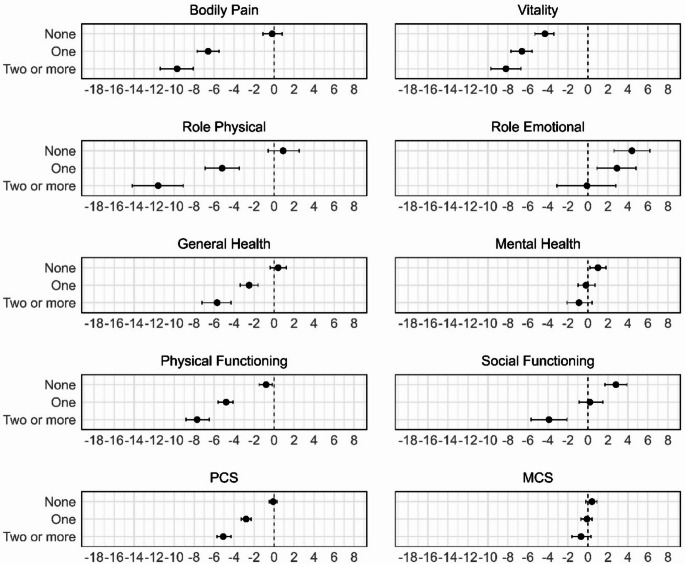
Adjusted change in mean score from Survey 1 to Survey 9 for women with no condition at baseline survey, stratified by number of conditions by Survey 9 (*N* = 9323). Results were adjusted for the effects of place of residence, marital status, education, income, smoking, alcohol consumption, physical activity, Body Mass Index (BMI), and menopause status

From Survey 1 to Survey 9, no change in PCS outcomes was observed among women without chronic conditions (Fig. 2). Decline in PCS outcomes were greater in women with multimorbidity than with one condition. MCS results were mixed. Most outcomes improved among women without chronic conditions, except for vitality, which declined. Role emotional did not change in those with multimorbidity but improved in women with no or only one condition. Vitality declined with increasing number of conditions.

**Fig. 3:**
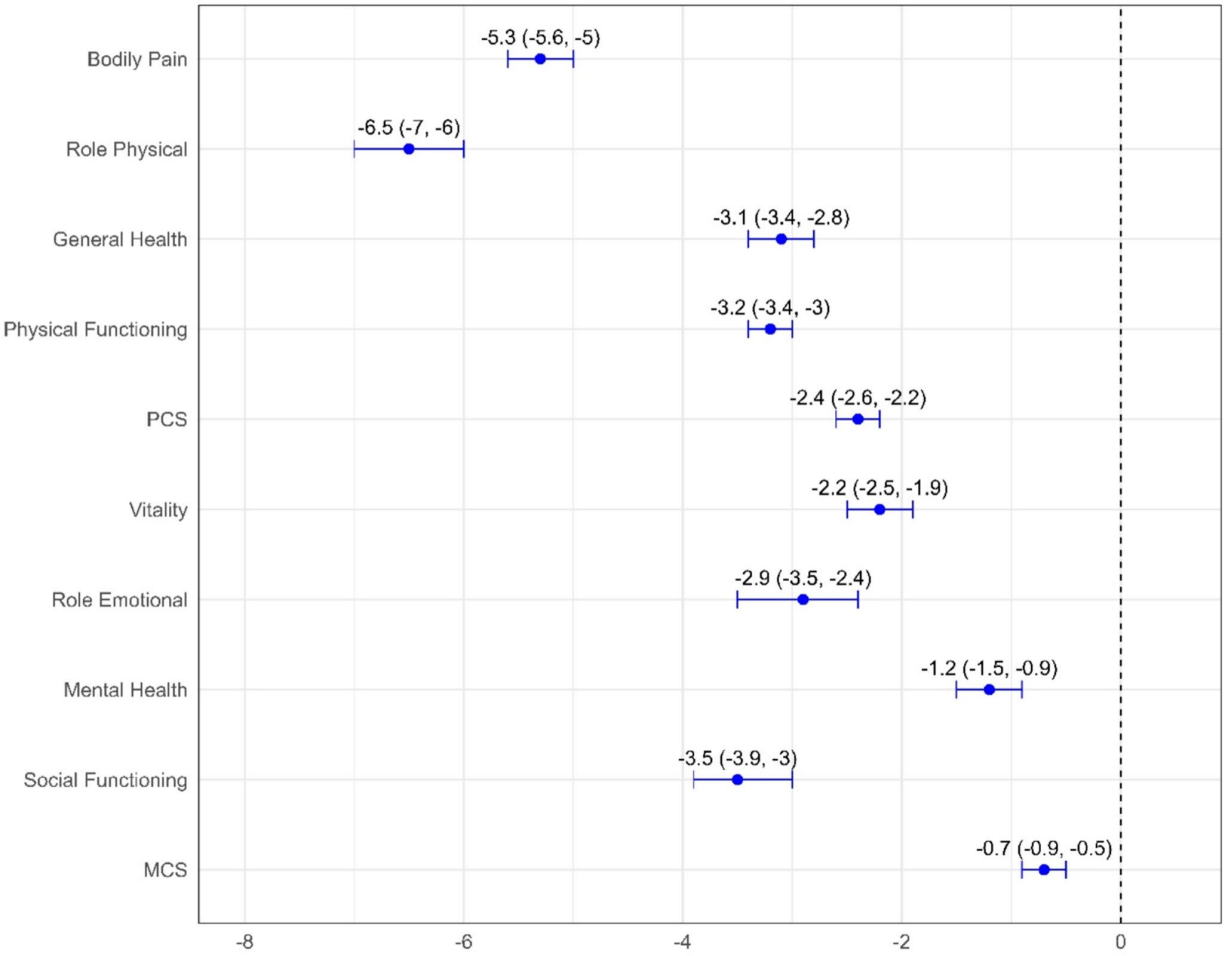
Reduction in outcomes per an additional chronic condition for women with no condition at baseline survey (*N* = 9323). Results were adjusted for the effects of place of residence, marital status, education, income, smoking, alcohol consumption, physical activity, Body Mass Index (BMI), and menopause status

Supplementary Table S2 shows a decline in the marginal means of outcomes with an increasing number of conditions. Outcomes reduced with each additional chronic condition (Fig. 3), with PCS outcomes being more affected than MCS outcomes. The largest average declines were observed in role physical and bodily pain. The mental health was less affected, with an average decline of 1.2 (0.9, 1.5) per additional condition.

For summary component scores, an extra condition was associated with a decline of 2.42 (2.27, 2.57) in PCS and a 0.76 (0.57, 0.97) in MCS summary scores (in Fig. 3).

Sociodemographic and lifestyle factors were also associated with HRQOL (Supplementary Tables S3 and S4). Women residing in inner and outer regional areas generally reported slightly higher HRQOL scores compared with those living in major cities. A greater ability to manage on income was associated with better scores. Health-related behaviours also showed consistent associations: physically active women reported higher HRQOL, whereas current smoking and obesity were associated with lower scores. Moreover, compared with premenopausal women, those who were peri- or postmenopausal reported lower scores.

Interactions were observed between the number of chronic conditions and physical activity, ability to manage on income, and BMI. Women with lower physical activity, greater financial difficulty, or obesity experienced larger reductions in HRQOL as the number of chronic conditions increased.

### Association between individual conditions and HRQOL

Supplementary Figs. S1 to S6 show the trajectories by condition. Supplementary Table S5 shows the estimated area between the trajectory curves from ages 18 and 49. Across all conditions, the effect was more pronounced for PCS than for MCS outcomes.

**Fig. 4:**
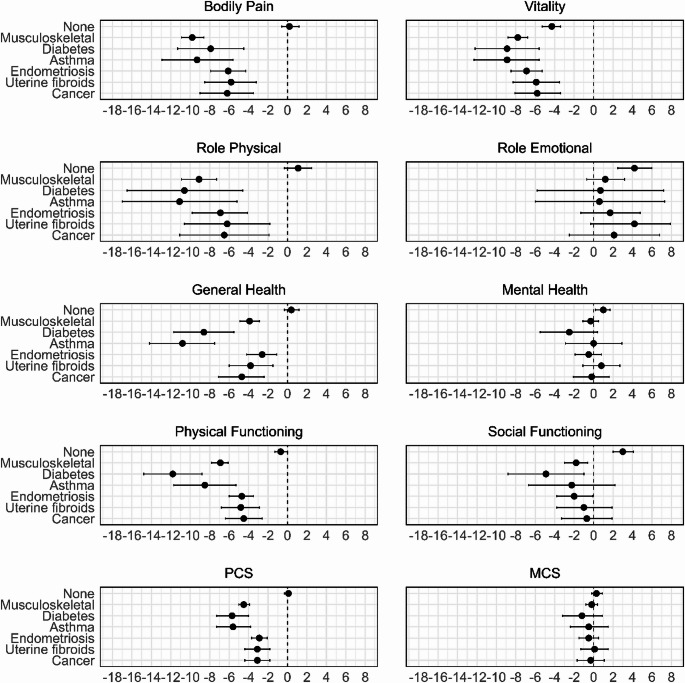
Average change in outcomes from survey 1 to 9 among women with each condition (*N* = 9323). Results were adjusted for the effects of place of residence, marital status, education, income, smoking, alcohol consumption, physical activity, Body Mass Index (BMI), and menopause status

Within-group analysis found that all six conditions were associated with a decline in PCS outcomes (Fig. 4). For MCS outcomes, none of the conditions was associated with a significant change in role emotional or mental health. In contrast, vitality declined across all groups.

**Fig. 5:**
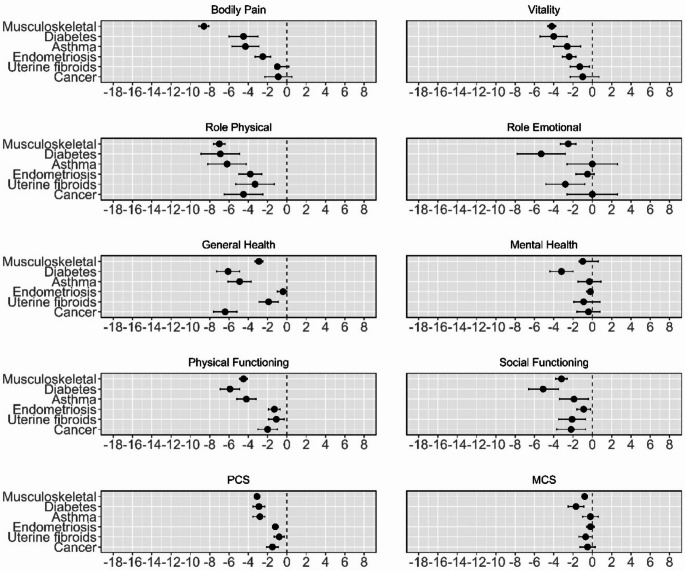
Difference in estimated marginal means and its 95% CI between women with and without a record of each condition (*N* = 9323). Results were adjusted for the effects of place of residence, marital status, education, income, smoking, alcohol consumption, physical activity, Body Mass Index (BMI), and menopause status

Supplementary Table S6 present the marginal means for women with and without each condition, with the mean differences and 95% CI. Between-group analysis showed that, compared to women without the same condition, women with musculoskeletal disorders, diabetes, or asthma had consistently lower scores across nearly all outcomes (Fig. 5), with the differences being greater for PCS than for MCS outcomes. Similar and smaller reductions were observed for endometriosis, uterine fibroids, and cancer (Fig. 5). In particular, cancer had a more profound effect on PCS than on MCS outcomes.

### Sensitivity analysis

 The main analysis used a missing category to handle the missing data. To assess the robustness of the findings (presented in Figs. 2, 3, 4 and 5), sensitivity analyses were conducted using multiply imputed data sets. Multiple imputation yielded estimates consistent with missing category analyses

## Discussion

Using nearly three decades of longitudinal data from a large population-based cohort of Australian women, this study examined the association between both the cumulative number and individual chronic conditions on HRQOL. Understanding these patterns is important as it offers insight for targeting early intervention and shaping preventive strategies. While clear longitudinal associations were observed, these findings do not imply causation and should be interpreted as temporal associations.

### Number of conditions

In this research, we used ‘number of chronic conditions’ to capture the overall association. Women who remained free of chronic conditions from Survey 1 to Survey 9 maintained stable PCS scores and improved MCS scores. In contrast, women who developed multimorbidity experienced substantial declines (especially in PCS dimensions), with smaller reductions among those with only one condition. Results for MCS outcomes varied: role emotional scores improved in those with no or one condition but remained unchanged in those with multimorbidity. Vitality declined across all groups, with the greatest reductions among those with multimorbidity. These results underscore the value of preventing chronic conditions in early and mid-adulthood for maintaining long-term physical and mental well-being.

To assess the overall burden of chronic conditions, some studies used multivariate approaches to group conditions based on their co-occurrence. Methods such as cluster analysis [22, 23] or latent class analysis [24, 25] have been used to identify homogeneous groups of conditions and examine their collective effects on HRQOL. Other studies focused on specific combinations of conditions (i.e., dyads or triads) [26, 27] or evaluated interaction effects between conditions [14, 28]. Given the relatively young age group of women in our cohort and the low prevalence of many conditions, these methods were not applied in the present study.

Our results also revealed that PCS scores were progressively lower among women with a higher number of chronic conditions. The analysis indicated that with each added condition, the PCS summary score decreased by 2.42 units (2.27, 2.57), while the MCS summary score declined by 0.76 units (0.57, 0.97).

A meta-analysis of 15 studies reported a reduction of 3.27 (1.74, 4.79) in PCS and 1.55 (0.13, 2.97) for MCS summary scores [11] per additional chronic condition, although differences in sample size, demographics, and study design limit comparison. Only four of the included studies had large sample sizes [15, 16, 29, 30]. Two of these large-scale studies focused on women [15, 31]. Luo et al. studied 75,198 women aged 50 to 79 at baseline and found that the onset of one, two, or three chronic conditions over three years was associated with PCS summary score declines of 2.5, 5.4, and 7.6 points, respectively, and MCS summary score declines of 1.1, 2.8, and 5.3 points [15]. Another study of 33,386 women aged 80 years and older found that compared to women with zero or one condition, scores were 8, 13, and 19 points lower among women with two, three, or four or more conditions [31]. Williams et al. analysed 23,789 participants (51.9% women) across a broad age range and found that having three or more chronic conditions was associated with 9.15 (8.61, 9.69) points lower PCS and 1.98 (1.52, 2.43) points lower MCS summary score [30]. Chin et al. studied 9028 participants (57.8% women; median age 48) attending primary care clinics and found that multimorbidity was associated with a 2.66 (2.44, 2.88) point decline in PCS summary score [29].

While our findings are consistent with these studies, this study provides important added value by revealing a clear gradual longitudinal association between the number of chronic conditions and HRQOL outcomes among women of reproductive age, an underrepresented group in multimorbidity research.

### Individual conditions

Within-group comparisons found that all six conditions were associated with a decline in physical health, as reflected by a reduction in PCS outcomes, with musculoskeletal disorders, diabetes, and asthma having the most profound effect. In contrast, for MCS, none of the outcomes resulted in a significant decline in role emotional or mental health. On the other hand, vitality declined across all six conditions, including women with none of these six conditions.

Our findings of condition and dimension-specific effects align with prior within-group longitudinal research. For example, Karlsen et al. compared the declines in HRQOL among women with and without breast cancer, and found that the mean difference in change was most pronounced in role physical, with the confidence interval for several other dimensions including the null value of zero, supporting a dimension-specific effect [32]. Similarly, another study reported a decline in physical health score among patients with various types of cancer, but mental health decline was observed only among patients with lung, colorectal, and prostate cancer [33].

Between-group comparisons in the present study showed that, compared to women without the same condition, all six conditions were associated with poorer HRQOL. However, the magnitude of the effect varied across outcomes. For example, musculoskeletal disorders, diabetes, and asthma were associated with poorer scores across almost all outcomes. Endometriosis, uterine fibroids, and cancer had a similar and smaller effect.

These findings are consistent with previous research. For example, a meta-analysis of six studies reported lower HRQOL scores across all dimensions among patients with osteoarthritis compared with healthy controls, especially in the score related to PCS outcomes [34]. Another study in a primary care setting found that patients with chronic musculoskeletal pain had lower HRQOL compared to patients without musculoskeletal pain [35]. Diabetes has also been associated with lower HRQOL [36]. In asthma, a study of 10,222 patients reported 5.3 points lower PCS (43.2 vs. 48.5) and 2.6 points lower MCS scores (48.4 vs. 51.0) compared to controls [37]. Longitudinal evidence also reported the negative effect of endometriosis on HRQOL, especially in dimensions related to PCS [38]. Similarly, women with symptomatic fibroids reported lower overall well-being compared to those without fibroids [39]. For cancer, evidence suggests a more dimension-specific effect. Helgesson et al. found that women with cancer had poorer general health compared to other women, while the confidence interval for the other dimensions covered the null value of zero [40]. The lack of association between mental health scores and cancer has been reported in the Iowa Women’s Health Study as well [41].

### Scoring properties of the SF-36 and implications for mental health findings

The pattern, where neither individual chronic conditions nor the number of conditions was associated with a decline in MCS (as shown in Figs. 4 and 2), may partly reflect properties of the SF-36 summary scoring approach rather than an absence of mental health impact. The SF-36 PCS and MCS are constructed to provide maximally independent measurement of physical and mental health, using an orthogonal factor solution that forces the two summary scores to be uncorrelated [42]. Consequently, when a condition primarily affects physical dimensions (e.g., role physical or general health), declines are captured strongly in PCS, while concurrent changes in the mental health domain that are correlated with physical health (e.g., role emotional or mental health) may be attenuated in MCS. In addition, the SF-36 mental health domains are designed as broad, generic indicators of psychological well-being and may be less sensitive to subtle psychological or emotional changes [43]. In support of this, Cunningham et al. showed that the RAND-36 questionnaire, which allows physical and mental health to be correlated, yielded larger mental health decrements than the SF-36 for each chronic condition, and noted that the SF-36 orthogonal approach can make it difficult to detect simultaneous physical and mental impacts even when both are affected [42].

### Content and construct validity of SF-36

Beyond scoring considerations, interpretation of our findings also requires careful attention to the construct and content validity of the SF-36 questionnaire. The SF-36 has been extensively validated and is commonly used to compare health status across populations and over time, supporting its construct validity as a generic measure of health-related quality of life [44].

With respect to content validity, the SF-36 is designed to assess HRQOL by capturing individuals’ self-reported physical and mental functioning and perceived health status across defined health domains. As a patient-reported outcome measure, it reflects individuals’ own perceptions of their functioning and well-being rather than clinicians’ evaluations of health status or healthcare performance. Moreover, HRQOL is distinct from broader notions of general quality of life, such as those articulated by the World Health Organisation, which encompass additional domains such as social participation, financial circumstances, and spirituality [45]. These broader domains are not captured by the SF-36, and therefore, the instrument does not provide a comprehensive assessment of overall quality of life. Accordingly, while HRQOL measures such as the SF-36 are informative, conclusions should remain aligned with this construct boundary.

### Clinical and policy-related implications

The findings that physical HRQOL was lower among women with a single chronic condition compared with those without any condition, together with the finding that each individual condition was associated with deterioration in physical HRQOL over time, suggests that physical functional decline occurs early in the chronic disease trajectory. These findings indicate that the impact is not confined to later stages characterised by the emergence of multiple chronic conditions. Clinically, this suggests that waiting until multimorbidity develops may be too late to prevent early functional deterioration. Multimorbidity frameworks predominantly emphasise enhanced management, care coordination, and service planning for individuals with multimorbidity [46]. In this context, reliance on multimorbidity as the primary threshold for heightened clinical or service attention may overlook declines in physical functioning among women living with a single diagnosed condition. While our study was not designed to develop risk stratification tools or clinical decision algorithms, these findings suggest that greater attention to physical functional status at the onset of chronic disease may be warranted within routine chronic disease management in primary care. Operationally, this may be supported by incorporating patient-reported outcome measures (PROM) of HRQOL into routine chronic condition management reviews, where PROM feedback at the point of care has been shown to improve detection of symptoms and aspects of clinician–patient communication [47].

The cultural and healthcare system context is also relevant, as HRQOL reflects not only underlying disease burden but also how health systems respond to health needs. These factors can influence access to health care and continuity of management [48]. Longitudinal evidence from Australia demonstrates that multimorbidity is associated with substantial reductions in HRQOL among Aboriginal and Torres Strait Islander peoples, and has been used as a key marker to prompt comprehensive and culturally sensitive health strategies [49]. In such settings, predominant focus on multimorbidity as a trigger for intensified service attention and coordinated care may miss earlier declines in physical HRQOL that occur with the onset of a single chronic condition. A life-course approach that integrates early functional assessment with culturally responsive care models may therefore strengthen preventive strategies.

From an intervention perspective, the conditions showing the largest decrements in physical HRQOL in our analyses (notably musculoskeletal disorders and diabetes) are also conditions for which evidence-based management strategies have demonstrated improvements in patient-reported outcomes, including quality of life. For chronic low back pain, a major component of the musculoskeletal disorder definition in this study, systematic review evidence indicates that exercise therapy improves pain and functional limitations compared with usual care or no treatment [50]. For diabetes, systematic review evidence indicates that structured diabetes self-management education/support can improve quality-of-life outcomes, reinforcing the relevance of early supportive management even before multimorbidity develops [51]. Taken together, physical HRQOL decrements observed with the onset of individual conditions are not only clinically meaningful but also potentially modifiable.

### Limitations

Our study had some limitations. First, there were differences in the periods when data were available for various chronic conditions (Supplementary Table S1). Second, although we adjusted the results for a range of sociodemographic and health-related covariates, residual confounding cannot be fully excluded and should be considered when interpreting the observed associations. Third, the diagnosis of chronic conditions was based on self-report (from ALSWH surveys) and administrative health records. However, the use of linked administrative datasets represents a major strength of this study. As summarised in Supplementary Table S1, multiple sources of data were used to identify reports of chronic conditions. Therefore, even if women were no longer completing the ALSWH surveys, the incidence of chronic conditions could be identified using other sources. Nevertheless, some cases may have been missed. Moreover, it should be acknowledged that the ALSWH experienced moderate nonresponse at consecutive surveys.

Another consideration relates to the accuracy of the self-reported conditions. Previous ALSWH studies demonstrated good validity for self-reported diagnosis, including for endometriosis [52], cancer [53], diabetes [54], and stroke [55]. While some misclassification may remain, the use of multiple data sources is likely to reduce overall misclassification.

Finally, given nearly 30 years of follow-up, attrition may have introduced bias. If women with poorer health (i.e., women with more chronic conditions and lower HRQOL) were more likely to discontinue participation, the observed associations may underestimate the strength of the association between chronic conditions and HRQOL. Although linked administrative data allowed ascertainment of incident conditions even when women did not complete later surveys, HRQOL outcomes relied on survey completion.

Despite these limitations, this is the first population-based study focused on women of reproductive age to track the longitudinal association between multimorbidity and HRQOL. Assessing the external validity beyond Australian women requires replication of similar studies in similarly aged cohorts in other countries and across diverse ethnic groups.

## Conclusions

This study highlights the association between chronic conditions and HRQOL among women of reproductive age. Women who remained free of chronic conditions throughout the follow-up maintained stable physical health and experienced improvements in mental well-being, underscoring the long-term benefits of prevention. In contrast, the development of multimorbidity was associated with a progressive decline in HRQOL, particularly in PCS. All six conditions were linked to reductions in PCS, with musculoskeletal disorders, diabetes, and asthma showing the most pronounced effects. MCS outcomes were more variable and appeared to be condition- and dimension-specific, with gynaecological conditions and cancer showing smaller and more specific effects. These findings support early intervention and prevention strategies focused on reducing the burden of chronic conditions to preserve HRQOL across the life course.

## Supplementary Information

Below is the link to the electronic supplementary material.

Supplementary Material 1

## Data Availability

The data that support the findings of this study are not openly available to protect the privacyof study participants.
