# Supplementary material for: Quality of life among women of reproductive age during the accumulation of multiple chronic conditions
Source: Qual Life Res. 2026 May 3;35(6):151. doi: 10.1007/s11136-026-04262-4 (PMC13136207; doi:10.1007/s11136-026-04262-4)
Supplement: Supplementary file 1 — Supplementary Material 1 [file 11136_2026_4262_MOESM1_ESM.docx]

**Supplementary materials**

| Table S1: List of conditions and data sources used (with coverage period) to identify patients | Page 3 |
| --- | --- |
| Table S2: Adjusted marginal means of domains of HRQOL outcomes for women with different numbers of conditions (N=9323) | Page 4 |
| Table S3: Adjusted associations between sociodemographic and health-related covariates and physical HRQOL domains | Pages 5 & 6 |
| Table S4: Adjusted associations between sociodemographic and health-related covariates and mental HRQOL domains | Pages 7 & 8 |
| Figure S1: Average score of dimensions of quality of life for women with no condition at baseline survey, stratified by musculoskeletal status at the end of the survey | Page 9 |
| Figure S2: Average score of dimensions of quality of life for women with no condition at baseline survey, stratified by endometriosis status at the end of the survey | Page 10 |
| Figure S3: Average score of dimensions of quality of life for women with no condition at baseline survey, stratified by uterine fibroids status at the end of the survey | Page 11 |
| Figure S4: Average score of dimensions of quality of life for women with no condition at baseline survey, stratified by cancer status at the end of the survey | Page 12 |
| Figure S5: Average score of dimensions of quality of life for women with no condition at baseline survey, stratified by diabetes status at the end of the survey | Page 13 |
| Figure S6: Average score of dimensions of quality of life for women with no condition at baseline survey, stratified by asthma status at the end of the survey | Page 14 |
| Table S5: Separation between two trajectory curves for each outcome based on reporting of each condition at the end of the survey | Page 15 |
| Table S6: Estimated marginal means of domains of HRQOL outcomes and their differences (95% CI) for each condition | Pages 16 & 17 |

**Sources of data and coverage periods**

Hospital and emergency department data were obtained from each State and Territory, with some variations in coverage dates between jurisdictions. Data on investigations and procedures (e.g., glycosylated haemoglobin tests or angioplasty) were obtained from the universal health insurance scheme, Medicare (for items listed on the Medical Benefits Schedule, MBS). Similarly, data on government-subsidised medications were obtained from the Pharmaceutical Benefits Scheme (PBS) which is available to all residents. Additional data on diagnoses were available from various assessments for government supported aged care. The causes of death, including underlying and contributing causes were obtained from multiple causes coded data.

The cancer data only included malignant cancers, so no skin cancer is included except for melanoma. Diabetes included type 1 and type 2 diabetes mellitus and excluded gestational diabetes. The asthma dataset was selected especially for asthma. The fibroids only included uterine fibroids. Musculoskeletal disorders included back pain, rheumatoid arthritis, osteoarthritis, ankylosing spondylitis, cervical disc displacement, sciatica, spinal deformities, and scoliosis.

Table S1: List of conditions and data sources used (with coverage period) to identify patients

|  | Cause of Death  1996 to 2019 | MBS^1^  1984 to 2021 | PBS^2^  2002 to 2021 | Hospital admissions/ emergency^3^, varied dates | Aged care^4^  , varied dates | ALSWH^5^  1996 to 2019 | Cancer registry |
| --- | --- | --- | --- | --- | --- | --- | --- |
| Cancer | ✓ | ✓ From 2018 |  | ✓ From 2018 |  |  | ✓ |
| Diabetes | ✓ | ✓ | ✓ | ✓ | ✓ | ✓ |  |
| Asthma | ✓ | ✓ | ✓ | ✓ |  | ✓ |  |
| Uterine fibroid | ✓ | ✓ | ✓ | ✓ |  | ✓ |  |
| Musculoskeletal  disorders | ✓ |  | ✓ | ✓ | ✓ | ✓ |  |
| Endometriosis |  | ✓ | ✓ | ✓ |  | ✓ |  |

^1^ MBS: Medicare Benefits Schedule; ^2^ PBS: Pharmaceutical Benefits Scheme; ^3^ coverage of hospital data varied between States and Territories, starting between 1970 and 2007, and ending between 2017 and 2021; ^4^ Coverage of aged care data varied between schemes, starting between 1997 and 2008 and ending between 2015 and 2020. The coverage for cancer registries varied from 1982-2008 to 2019.

^5^The first and last ALSWH surveys used in this study were conducted in 1996 and 2019.

Table S2: Adjusted marginal means of domains of HRQOL outcomes for women with different numbers of conditions (N=9323)

|  | **0** | **1** | **2** | **≥3** |
| --- | --- | --- | --- | --- |
| Bodily pain | 75.7 (75.2, 76.2) | 69.4 (68.8, 70.0) | 65.5 (64.4, 66.3) | 62.5 (61.0, 64.7) |
| Role physical | 83.4 (82.6, 84.1) | 76.8 (75.9, 77.7) | 70.7 (69.2, 72.2) | 62.6 (59.8, 65.4) |
| General health | 70.8 (70.3, 71.2) | 67.8 (67.2, 68.4) | 64.6 (63.7, 65.4) | 61.2 (59.6, 62.8) |
| Physical functioning | 89.9 (88.6, 90.3) | 86.7 (86.2, 87.1) | 83.6 (82.8, 84.3) | 80.1 (78.8, 81.4) |
| PCS | 49.1 (48.4, 49.2) | 46.5 (46.2, 46.7) | 44.3 (43.9, 44.8) | 42.1 (41.2, 42.8) |
| Role emotional | 76.2 (75.3, 77.1) | 73.6 (72.5, 74.6) | 70.0 (68.3, 71.7) | 66.5 (63.4, 69.6) |
| Mental health | 69.8 (69.3, 70.3) | 68.6 (68.0, 69.1) | 67.3 (66.5, 68.1) | 66.5 (65.1, 68.0) |
| Social functioning | 79.7 (79.1, 80.2) | 76.3 (75.7, 77.0) | 72.4 (71.3, 73.5) | 69.3 (67.3, 71.3) |
| Vitality index | 53.9 (53.4, 54.4) | 51.5 (50.9, 52.1) | 49.3 (48.4, 50.3) | 47.5 (45.8, 49.2) |
| MCS | 46.4 (46.1, 46.8) | 45.7 (45.4, 46.1) | 44.8 (44.2, 45.3) | 44.2 (43.2, 45.2) |

Results are adjusted for time-dependent effects of place of residence, marital status, education, income, smoking, alcohol, physical activity, Body Mass Index (BMI), and menopause status.

Higher scores indicate better HRQOL.

Table S3: Adjusted associations between sociodemographic and health-related covariates and physical HRQOL domains

| Variable | Category | Physical Functioning | Role Physical | Bodily Pain | General Health |
| --- | --- | --- | --- | --- | --- |
| area of residence | Major cities | Reference | Reference | Reference | Reference |
|  | Inner regional | 0.32 (0.01, 0.63) | 1.02 (0.35, 1.69) | 0.17 (-0.26, 0.60) | 0.60 (0.21, 0.99) |
|  | Outer regional | 0.53 (0.12, 0.94) | 1.76 (0.88, 2.64) | 0.56 (-0.03, 1.15) | 0.99 (0.48, 1.50) |
|  | Remote | 0.82 (0.04, 1.60) | 1.28 (-0.43, 2.99) | 1.16 (0.04, 2.28) | 1.86 (0.92, 2.80) |
| marital status | Married | Reference | Reference | Reference | Reference |
|  | S/D/W | 0.44 (-0.07, 0.95) | 1.38 (0.22, 2.54) | 0.80 (0.07, 1.53) | 0.33 (-0.26, 0.92) |
|  | Single | 0.29 (-0.02, 0.60) | 1.30 (0.63, 1.97) | 0.29 (-0.14, 0.72) | -0.52 (-0.87, -0.17) |
| education level | Below diploma | Reference | Reference | Reference | Reference |
|  | Diploma | 0.19 (-0.16, 0.54) | 0.02 (-0.76, 0.80) | -0.13 (-0.64, 0.38) | 0.07 (-0.34, 0.48) |
|  | University degree | 0.92 (0.57, 1.27) | -0.21 (-0.97, 0.55) | 0.67 (0.18, 1.16) | -0.24 (-0.67, 0.19) |
| BMI | Normal | Reference | Reference | Reference | Reference |
|  | Underweight | 0.11 (-0.54, 0.76) | 1.54 (0.27, 2.81) | 0.54 (-0.36, 1.44) | -0.11 (-1.13, 0.91) |
|  | Overweight | -1.91 (-2.42, -1.40) | -0.07 (-1.09, 0.95) | -0.88 (-1.61, -0.15) | -3.40 (-4.22, -2.58) |
|  | Obesity | -5.81 (-6.59, -5.03) | -1.35 (-2.90, 0.20) | -2.88 (-3.98, -1.78) | -8.40 (-9.63, -7.17) |
| alcohol consumption | Low | Reference | Reference | Reference | Reference |
|  | None | -4.10 (-4.53, -3.67) | -6.87 (-7.81, -5.93) | -1.70 (-2.29, -1.11) | -0.49 (-1.00, 0.02) |
|  | Rare | -1.54 (-1.81, -1.27) | -3.36 (-3.97, -2.75) | -1.24 (-1.61, -0.87) | -0.80 (-1.11, -0.49) |
|  | Risky/ very risky | -0.34 (-0.83, 0.15) | 0.64 (-0.44, 1.72) | 0.10 (-0.59, 0.79) | -1.73 (-2.26, -1.20) |
| physical activity | Nil/ sedentary | Reference | Reference | Reference | Reference |
|  | Low | 4.57 (4.18, 4.96) | 6.79 (5.93, 7.65) | 3.66 (3.11, 4.21) | 2.53 (2.12, 2.94) |
|  | Moderate | 6.33 (5.92, 6.74) | 9.59 (8.67, 10.51) | 5.02 (4.43, 5.61) | 4.50 (4.05, 4.95) |
|  | High | 7.47 (7.06, 7.88) | 12.21 (11.31, 13.11) | 5.98 (5.41, 6.55) | 6.60 (6.15, 7.05) |
| smoking | Never | Reference | Reference | Reference | Reference |
|  | Ex | -0.97 (-1.32, -0.62) | -2.18 (-2.92, -1.44) | -1.47 (-1.96, -0.98) | -0.74 (-1.17, -0.31) |
|  | Current | -1.28 (-1.67, -0.89) | -2.08 (-2.88, -1.28) | -1.74 (-2.27, -1.21) | -4.68 (-5.17, -4.19) |
| manage on income | Impossible/ very difficult | Reference | Reference | Reference | Reference |
|  | Sometimes difficult | 1.54 (1.17, 1.91) | 2.77 (1.95, 3.59) | 1.91 (1.40, 2.42) | 2.26 (1.85, 2.67) |
|  | Not bad | 2.21 (1.84, 2.58) | 4.94 (4.12, 5.76) | 3.26 (2.75, 3.77) | 3.73 (3.32, 4.14) |
|  | Easy | 2.35 (1.92, 2.78) | 5.92 (4.98, 6.86) | 4.39 (3.78, 5.00) | 4.67 (4.18, 5.16) |
| menopause status | Premenopausal | Reference | Reference | Reference | Reference |
|  | Oophorectomy | -0.75 (-1.95, 0.45) | -4.20 (-6.85, -1.55) | -1.36 (-3.03, 0.31) | -0.47 (-1.86, 0.92) |
|  | Hysterectomy | -1.46 (-2.64, -0.28) | -4.39 (-7.04, -1.74) | -0.08 (-1.75, 1.59) | -0.32 (-1.69, 1.05) |
|  | Unknown due to HRT | -3.20 (-5.71, -0.69) | -7.75 (-13.43, -2.07) | -4.52 (-8.05, -0.99) | -4.88 (-7.64, -2.12) |
|  | Unknown due to contraceptive use | 2.00 (1.55, 2.45) | -0.10 (-1.14, 0.94) | 0.31 (-0.34, 0.96) | -0.63 (-1.14, -0.12) |
|  | Perimenopausal | -2.14 (-2.83, -1.45) | -4.41 (-5.96, -2.86) | -0.83 (-2.24, 0.58) | -2.51 (-3.25, -1.77) |
|  | Postmenopausal | -1.71 (-2.71, -0.71) | -5.40 (-7.63, -3.17) | -0.04 (-3.22, 3.14) | -2.47 (-3.63, -1.31) |

Legend: Values represent adjusted regression coefficients with 95% confidence intervals derived from linear mixed-effects models. Coefficients represent the mean difference in SF-36 physical HRQOL domain scores compared with the reference category. Positive coefficients indicate higher HRQOL, and negative coefficients indicate lower HRQOL

Table S4: Adjusted associations between sociodemographic and health-related covariates and mental HRQOL domains

| Variable | Category | Social Functioning | Role Emotional | Mental Health | Vitality |
| --- | --- | --- | --- | --- | --- |
| area of residence | Major cities | Reference | Reference | Reference | Reference |
|  | Inner regional | 1.05 (0.56, 1.54) | 1.11 (0.33, 1.89) | 0.58 (0.23, 0.93) | 0.54 (0.13, 0.95) |
|  | Outer regional | 0.92 (0.29, 1.55) | 1.21 (0.19, 2.23) | 0.75 (0.26, 1.24) | 1.37 (0.80, 1.94) |
|  | Remote | 1.28 (0.06, 2.50) | 1.88 (-0.06, 3.82) | 1.21 (0.31, 2.11) | 1.41 (0.35, 2.47) |
| marital status | Married | Reference | Reference | Reference | Reference |
|  | S/D/W | -4.55 (-5.33, -3.77) | -7.56 (-8.83, -6.29) | -2.79 (-3.36, -2.22) | 0.06 (-0.59, 0.71) |
|  | Single | -2.91 (-3.38, -2.44) | -4.38 (-5.12, -3.64) | -1.82 (-2.15, -1.49) | 1.59 (1.20, 1.98) |
| education level | Below diploma | Reference | Reference | Reference | Reference |
|  | Diploma | -0.73 (-1.28, -0.18) | -0.78 (-1.66, 0.10) | -0.27 (-0.66, 0.12) | -0.49 (-0.96, -0.02) |
|  | University degree | -0.00 (-0.55, 0.55) | -0.35 (-1.23, 0.53) | -0.34 (-0.75, 0.07) | -1.49 (-1.96, -1.02) |
| BMI | Normal | Reference | Reference | Reference | Reference |
|  | Underweight | -1.01 (-2.01, -0.01) | -0.21 (-1.80, 1.38) | -1.19 (-2.07, -0.31) | -0.45 (-1.47, 0.57) |
|  | Overweight | -0.43 (-1.23, 0.37) | -0.11 (-1.38, 1.16) | -0.62 (-1.33, 0.09) | -1.45 (-2.27, -0.63) |
|  | Obesity | -0.93 (-2.15, 0.29) | -0.37 (-2.31, 1.57) | -1.58 (-2.64, -0.52) | -2.85 (-4.08, -1.62) |
| alcohol consumption | Low | Reference | Reference | Reference | Reference |
|  | None | -1.84 (-2.51, -1.17) | 0.57 (-0.49, ,1.63) | 0.46 (-0.01, 0.93) | -1.96 (-2.51, -1.41) |
|  | Rare | -1.17 (-1.58, -0.76) | 0.07 (-0.60, 0.74) | -0.22 (-0.51, 0.07) | -1.35 (-1.68, -1.02) |
|  | Risky/ very risky | -1.62 (-2.35, -0.89) | -4.21 (-5.41, -3.01) | -2.15 (-2.66, -1.64) | -0.76 (-1.35, -0.17) |
| physical activity | Nil/ sedentary | Reference | Reference | Reference | Reference |
|  | Low | 4.52 (3.93, 5.11) | 1.77 (0.81, 2.73) | 1.34 (0.93, 1.75) | 3.53 (3.06, 4.00) |
|  | Moderate | 6.13 (5.50, 6.76) | 2.52 (1.50, 3.54) | 2.20 (1.77, 2.63) | 6.30 (5.79, 6.81) |
|  | High | 7.55 (6.94, 8.16) | 3.68 (2.68, 4.68) | 3.10 (2.67, 3.53) | 9.08 (8.57, 9.59) |
| smoking | Never | Reference | Reference | Reference | Reference |
|  | Ex | -2.17 (-2.70, -1.64) | -2.58 (-3.44, -1.72) | -0.97 (-1.38, -0.56) | -1.01 (-1.48, -0.54) |
|  | Current | -4.01 (-4.60, -3.42) | -7.31 (-8.25, -6.37) | -3.16 (-3.61, -2.71) | -2.38 (-2.91, -1.85) |
| manage on income | Impossible/ very difficult | Reference | Reference | Reference | Reference |
|  | Sometimes difficult | 4.21 (3.66, 4.76) | 6.06 (5.16, 6.96) | 3.54 (3.15, 3.93) | 3.02 (2.57, 3.47) |
|  | Not bad | 6.31 (5.74, 6.88) | 9.72 (8.82, 10.62) | 5.17 (4.78, 5.56) | 4.86 (4.39, 5.33) |
|  | Easy | 7.42 (6.77, 8.07) | 10.81 (9.75, 11.87) | 6.40 (5.93, 6.87) | 6.51 (5.98, 7.04) |
| menopause status | Premenopausal | Reference | Reference | Reference | Reference |
|  | Oophorectomy | -2.04 (-3.84, -0.24) | -1.47 (-4.37, 1.43) | -0.13 (-1.44, 1.18) | -1.58 (-3.09, -0.07) |
|  | Hysterectomy | -3.93 (-5.73, -2.13) | -3.90 (-6.80, -1.00) | -1.56 (-2.85, -0.27) | -1.23 (-2.72, 0.26) |
|  | Unknown due to HRT | -8.41 (-12.23, -4.59) | -8.05 (-14.20, -1.90) | -3.08 (-5.75, -0.41) | -4.42 (-7.52, -1.32) |
|  | Unknown due to contraceptive use | -0.52 (-1.23, 0.19) | -1.06 (-2.20, 0.08) | -0.82 (-1.31, -0.33) | -1.05 (-1.62, -0.48) |
|  | Perimenopausal | -2.92 (-3.96, -1.88) | -3.54 (-5.21, -1.87) | -1.86 (-2.59, -1.13) | -2.86 (-3.70, -2.02) |
|  | Postmenopausal | -3.74 (-5.27, -2.21) | -2.05 (-4.50, 0.40) | -1.17 (-3.72, 1.38) | -1.69 (-2.94, -0.44) |

Legend: Values represent adjusted regression coefficients with 95% confidence intervals derived from linear mixed-effects models. Coefficients represent the mean difference in SF-36 mental HRQOL domain scores compared with the reference category. Positive coefficients indicate higher HRQOL, and negative coefficients indicate lower HRQOL.

Figure S1: Average score of dimensions of quality of life for women with no condition at baseline survey, stratified by **musculoskeletal** status at the end of the survey


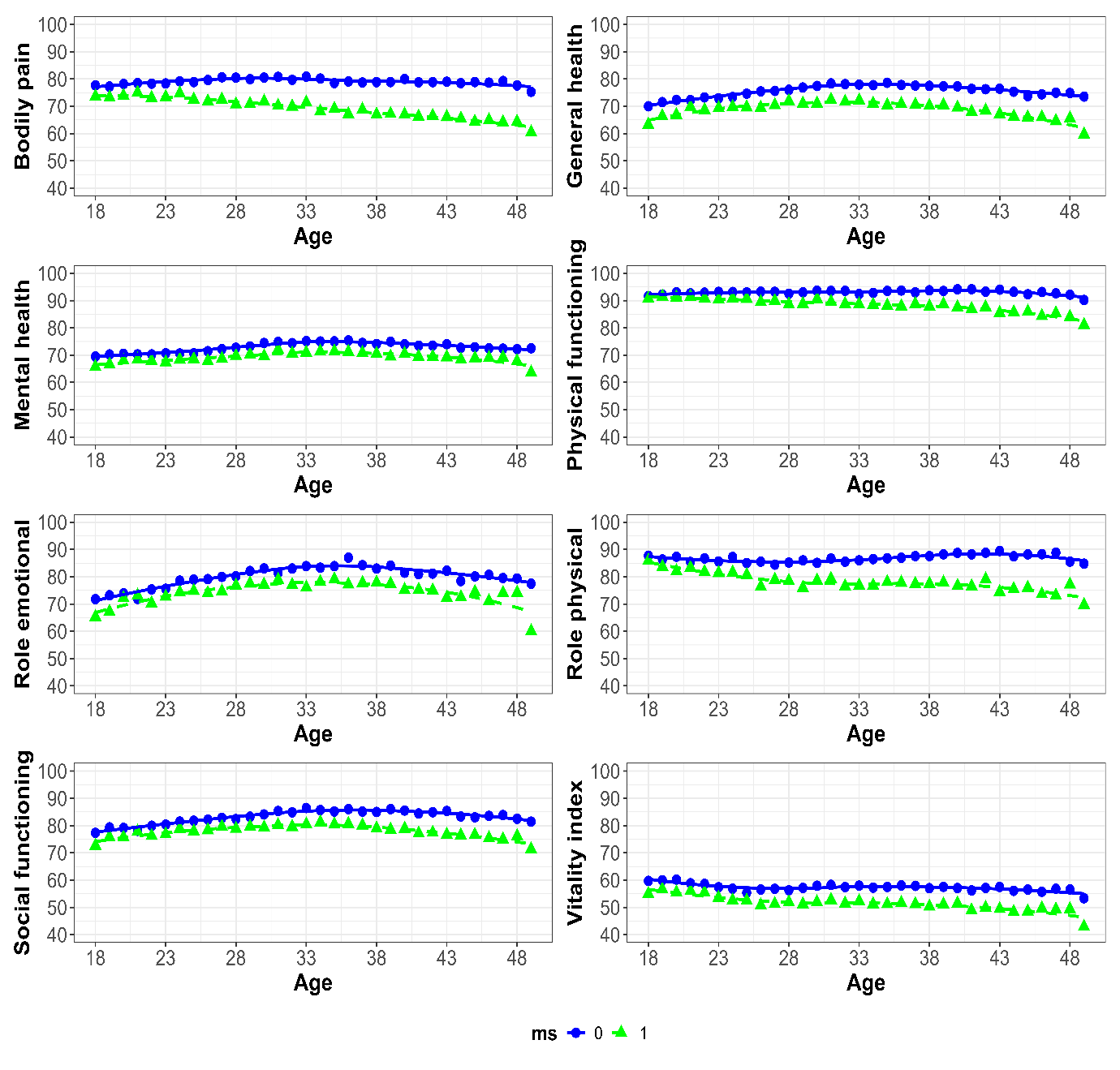


Legend for Figure S1: Higher scores indicate better HRQOL.

Figure S2: Average score of dimensions of quality of life for women with no condition at baseline survey, stratified by **endometriosis** status at the end of the survey


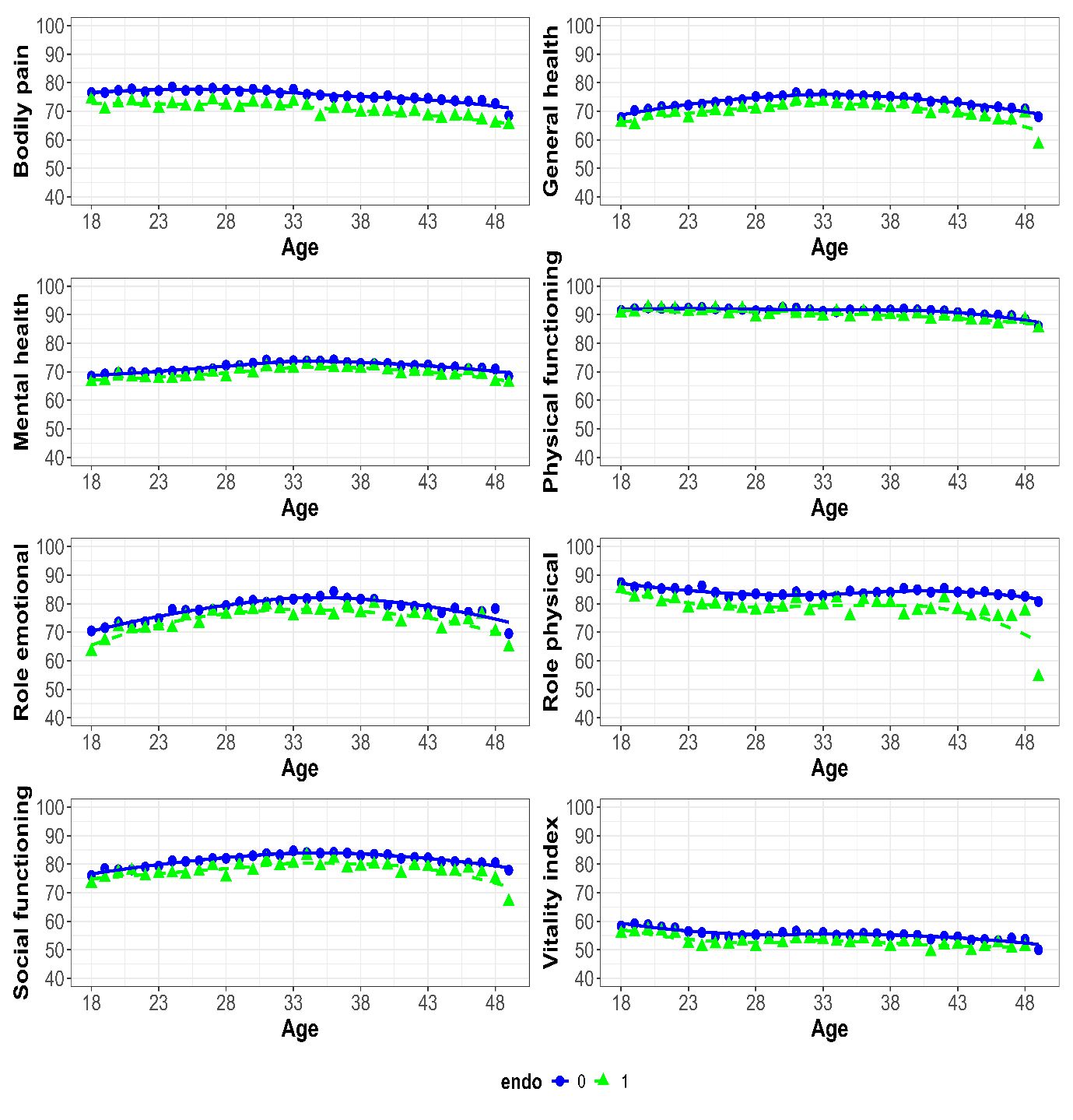


Legend for Figure S2: Higher scores indicate better HRQOL.

Figure S3: Average score of dimensions of quality of life for women with no condition at baseline survey, stratified by **uterine fibroids** status at the end of the survey


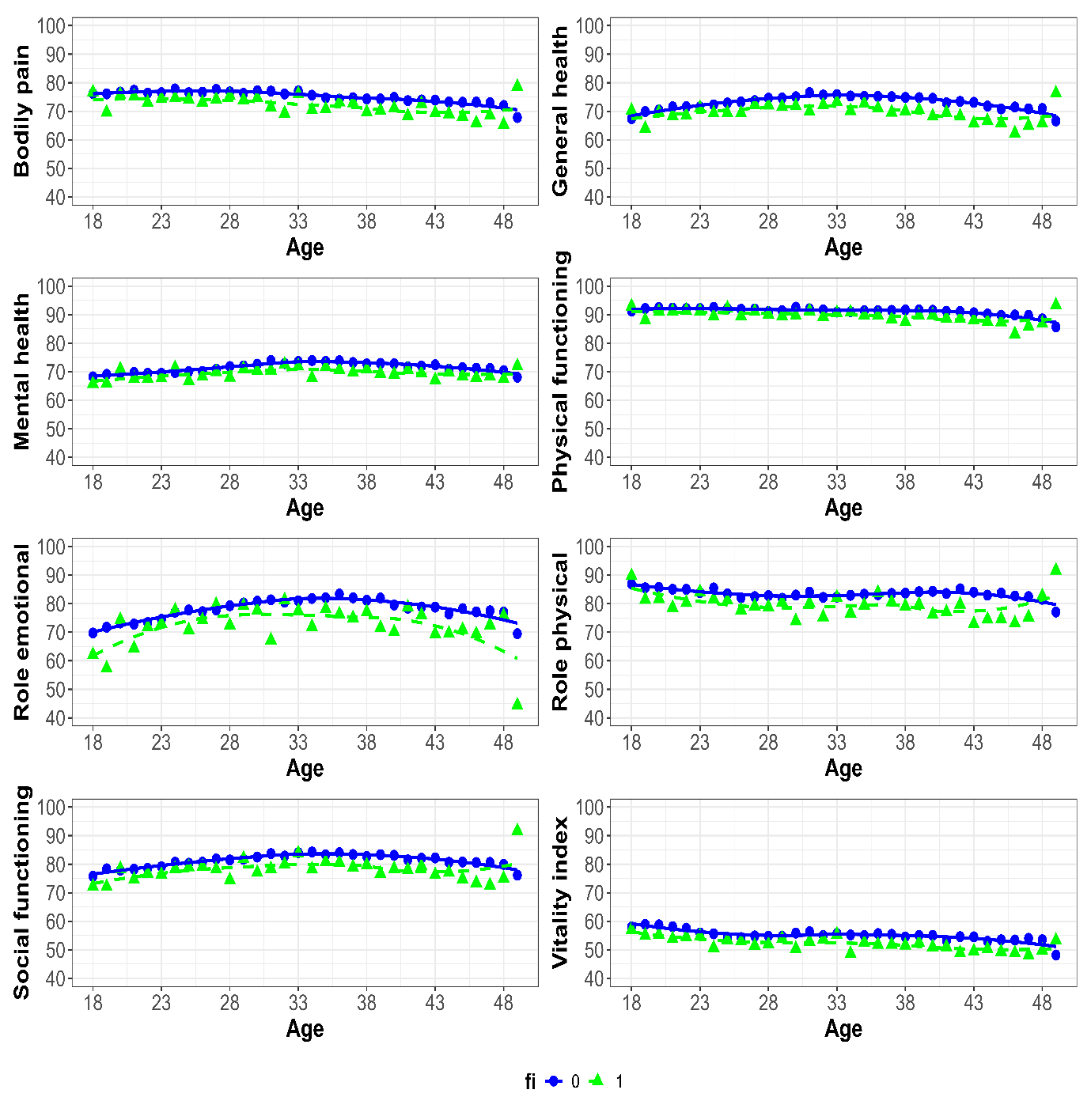


Legend for Figure S3: Higher scores indicate better HRQOL.

Figure S4: Average score of dimensions of quality of life for women with no condition at baseline survey, stratified by **cancer** status at the end of the survey


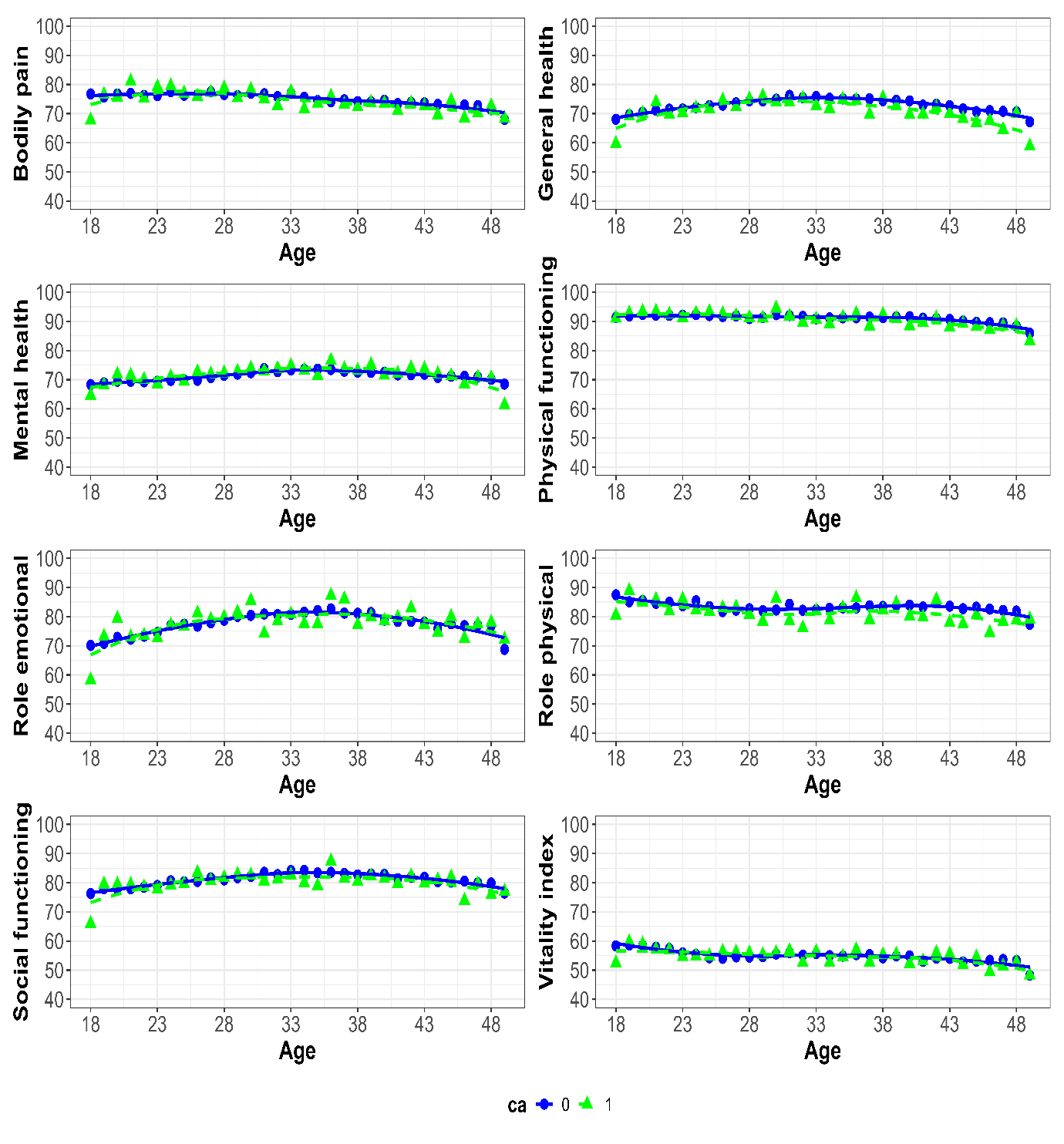


Legend for Figure S4: Higher scores indicate better HRQOL.

Figure S5: Average score of dimensions of quality of life for women with no condition at baseline survey, stratified by **diabetes** status at the end of the survey


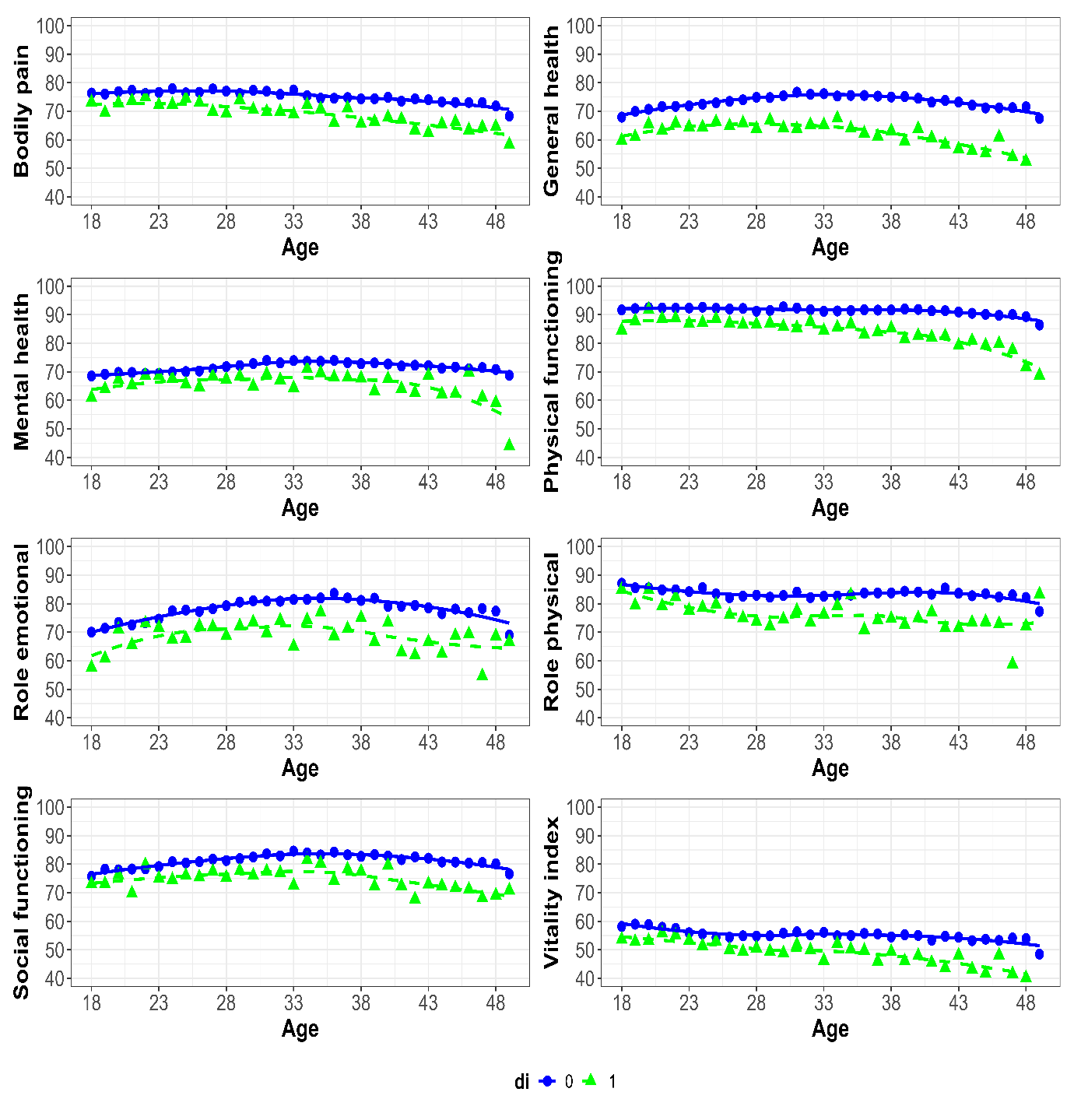


Legend for Figure S5: Higher scores indicate better HRQOL.

Figure S6: Average score of dimensions of quality of life for women with no condition at baseline survey, stratified by **asthma** status at the end of the survey


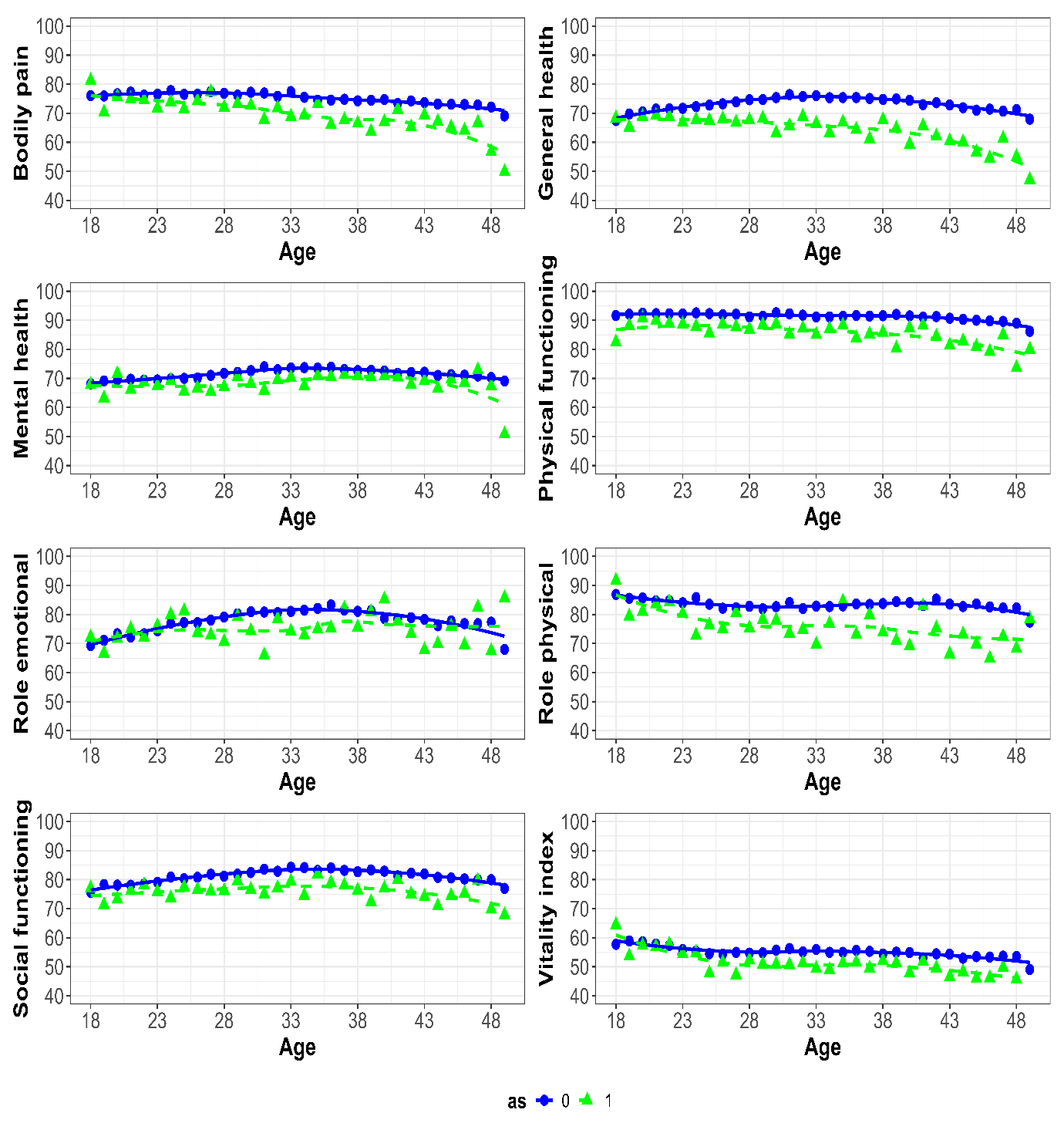


Legend for Figure S6: Higher scores indicate better HRQOL.

Table S5: Separation between two trajectory curves for each outcome based on reporting of each condition at the end of the survey

|  | Musculoskeletal | Diabetes | Asthma | Endometriosis | Fibroids | Cancer |
| --- | --- | --- | --- | --- | --- | --- |
| Bodily pain | 303.6 | 195.7 | 176.5 | 160.8 | 104.5 | 16.8 |
| Role physical | 271.4 | 244.1 | 237.7 | 166.8 | 134.8 | 55.8 |
| General health | 207.6 | 357.1 | 278.5 | 105.0 | 119.1 | 54.1 |
| Physical functioning | 145.8 | 226.3 | 179.1 | 41.0 | 57.4 | 11.7 |
| Role emotional | 174.1 | 295.9 | 92.5 | 115.1 | 167.8 | 6.4 |
| Mental health | 113.0 | 181.1 | 96.2 | 67.4 | 68.9 | 15.2 |
| Social functioning | 164.2 | 206.9 | 165.3 | 110.1 | 109.2 | 27.8 |
| Vitality index | 180.8 | 201.1 | 135.3 | 90.6 | 97.6 | 6.2 |

The area between trajectory curves between ages 18 to 49 were calculated to estimate the overall separation between trajectory curves

Table S6: Estimated marginal means of domains of HRQOL outcomes and their differences (95% CI) for each condition

|  | Musculoskeletal | Diabetes | Asthma | Endometriosis | Uterine fibroids | Cancer |
| --- | --- | --- | --- | --- | --- | --- |
| Bodily pain | 76.6 (76.4, 76.9)  68.0 (67.5, 68.5)  8.6 (8.1, 9.1) | 75.3 (75.0, 75.5)  70.8 (69.3, 72.3)  4.5 (3.0, 6.0) | 75.3 (75.0, 75.5)  70.9 (69.5, 72.5)  4.3 (2.9, 5.7) | 75.4 (75.1, 75.6)  72.8 (72.1, 73.6)  2.5 (1.7, 3.3) | 75.2 (75.0, 75.5)  74.2 (72.9, 75.5)  1.0 (-0.2, 1.2) | 75.2 (75.0, 75.5)  74.3 (72.8, 75.8)  0.9 (-0.5, 2.3) |
| Role physical | 84.2 (83.8, 84.5)  77.2 (76.4, 77.9)  7.0 (6.4, 7.6) | 83.1 (82.8, 83.4)  76.3 (74.0, 78.7)  6.9 (4.9, 8.9) | 83.1 (82.7, 83.4)  76.9 (74.6, 79.2)  6.2 (4.2, 8.2) | 83.3 (82.9, 83.6)  79.5 (78.3, 80.6)  3.8 (2.6, 5.0) | 83.1 (82.7, 83.4)  79.8 (77.8, 81.7)  3.3 (1.3, 5.3) | 83.1 (82.7, 83.4)  78.2 (75.9, 80.6)  4.5 (2.5, 6.5) |
| General health | 73.1 (72.8, 73.4)  70.1 (69.6, 70.6)  2.9 (2.5, 3.3) | 72.7 (72.4, 73.0)  66.5 (65.2, 67.9)  6.1 (4.9, 7.3) | 72.7 (72.4, 72.9)  67.8 (66.4, 69.1)  4.9 (3.7, 6.1) | 72.7 (72.3, 72.9)  72.3 (71.5, 72.9)  0.4 (-0.2, 1.0) | 72.7 (72.7, 72.9)  70.7 (69.6, 71.8)  1.9 (0.9, 2.9) | 72.7 972.4, 73.0)  66.3 (64.9, 67.6)  6.4 (5.2, 7.6) |
| Physical functioning | 91.8 (91.7, 92.0)  87.3 (87.0, 87.7)  4.5 (4.1, 4.9) | 91.2 (91.0, 91.4)  85.2 (84.1, 86.3)  5.9 (4.9, 6.9) | 91.1 (91.0, 91.4)  86.9 (85.8, 88.1)  4.2 (3.2, 5.2) | 91.2 (91.0, 91.4)  89.8 (89.3, 90.4)  1.3 (0.7, 1.9) | 91.1 (90.9, 91.3)  90.2 (89.3, 91.2)  0.9 (0, 1.9) | 91.1 (90.9, 91.3)  89.1 (88.0, 90.2)  2.0 (1.0, 3.0) |
| Physical component summary score | 49.5 (49.4, 49.6)  46.4 (46.1, 46.6)  3.2 (3.0, 3.4) | 48.9 (48.8, 49.1)  46.0 (45.4, 46.7)  2.9 (2.3, 3.5) | 48.9 (48.8, 49.1)  46.1 (45.1, 47.2)  2.8 (1.8, 3.8) | 49.0 (48.9, 49.2)  47.8 (47.5, 48.2)  1.1 (0.9, 1.4) | 48.9 (48.8, 49.1)  48.1 (46.9, 48.4)  0.8 (0.4, 1.2) | 48.9 (48.8, 49.1)  47.5 (46.8, 48.2)  1.4 (0.8, 2.0) |
| Role emotional | 78.2 (77.7, 78.7)  75.7 (74.9, 76.5)  2.5 (1.7, 3.3) | 77.9 (77.4, 78.3)  72.5 (69.9, 75.1)  5.3 (2.8, 7.8) | 77.8 (77.3, 78.2)  77.8 (75.1, 80.4)  0.0 (-2.6, 2.6) | 77.8 (77.4, 78.3)  77.4 (76.0, 78.7)  0.5 (-0.2, 1.7) | 77.8 (77.4, 78.3)  75.7 (73.5, 78.0)  2.1 (0.8, 4.8) | 77.8 (77.3, 78.2)  77.7 (75.1, 80.4)  0.0 (-2.6, 2.6) |
| Mental health | 71.2 (71.0, 71.5)  70.2 (69.8, 70.6)  1.0 (0.6, 1.4) | 71.1 (70.9, 71.4)  68.0 (66.7, 69.2)  3.2 (2.0, 4.4) | 71.1 (70.8, 71.3)  70.8 (69.5, 72.0)  0.3 (-0.9, 1.5) | 71.1 (70.8, 71.3)  71.1 (70.4, 71.7)  0.0 (-0.6, 0.6) | 71.1 (70.8, 71.3)  70.5 (69.4, 71.5)  0.6 (-0.4, 1.6) | 71.1 (70.8, 71.3)  70.7 (69.4, 71.9)  0.4 (-0.8, 1.6) |
| Social functioning | 81.3 (81.0, 81.6)  78.1 (77.5, 78.6)  3.2 (2.6, 3.8) | 80.8 (80.5, 81.1)  75.7 (74.0, 77.3)  5.1 (3.5, 6.6) | 80.8 (80.5, 81.0)  78.8 (77.1, 80.5)  1.9 (0.4, 3.4) | 80.8 (80.5, 81.1)  79.8 (79.0, 80.7)  0.9 (0.2, 1.6) | 80.8 (80.5, 81.1)  78.6 (77.2, 80.0)  2.1 (0.7, 3.5) | 80.8 (80.5, 81.1)  78.6 (76.9, 80.2)  2.2 (0.7, 3.7) |
| Vitality index | 55.8 (55.5, 56.1)  51.6 (51.1, 52.1)  4.2 (3.8, 4.6) | 55.2 (54.9, 55.4)  51.2 (49.7, 52.6)  4.0 (2.6, 5.4) | 55.1 (54.9, 55.4)  52.5 (51.1, 54.0)  2.6 (1.2, 4.0) | 55.3 (55.0, 55.5)  52.8 (52.1, 53.6)  2.4 (1.7, 3.1) | 55.1 (54.8, 55.4)  53.8 (52.6, 55.0)  1.3 (0.3, 2.3) | 55.1 (54.8, 55.4)  54.1 (52.7, 55.6)  1.0 (-0.7, 2.3) |
| Mental component summary score | 47.7 (47.5, 47.9)  46.9 (46.6, 47.2)  0.8 (0.6, 1.0) | 47.5 (47.4, 47.7)  45.9 (44.7, 47.1)  1.6 (0.5, 2.7) | 47.5 (47.3, 47.7)  47.3 (46.5, 48.3)  0.2 (-0.5, 0.9) | 47.5 (47.3, 47.7)  47.2 (46.6, 47.8)  0.3 (-0.3, 0.9) | 47.5 (47.4, 47.7)  46.8 (46.1, 47.5)  0.7 (0.0, 1.4) | 47.5 (47.3, 47.7)  47.5 (46.2, 47.9)  0.5 (-0.3, 1.3) |

The first and second rows show the estimated marginal means for women without and with the condition. The third row shows the difference in marginal means adjusted for the time-dependent self-reported effects of place of residence, marital status, education, income, smoking, alcohol, physical activity, and Body Mass Index (BMI); as well as the time-dependent effect of other chronic conditions.

Higher mean scores indicate better HRQOL.
